# Supplementary material for: Inhibition of Cancer Cell Migration and Invasion In Vitro by Recombinant Tyrosine-Sulfated Haemathrin, A Thrombin Inhibitor
Source: Int J Mol Sci. 2024 Nov 4;25(21):11822. doi: 10.3390/ijms252111822 (PMC11546549; doi:10.3390/ijms252111822)
Supplement: Supplementary file 1 [file ijms-25-11822-s001.zip › Supplementary figure captions.pdf]

Supplementary figure S1. The effect of sulfated haemathrin on the migration of SKOV3 and MDA-MB-231 cells using a wound-healing assay. The average distance between the edges of the gap was measured in three independent experiments. Haemathrin 2S inhibited thrombin-induced migration with statistical significance more effectively than haemathrin WT. Results are presented as mean +/- standard deviation of three independent experiments (*$p < 0.05$). SF, serum free; Haemathrin WT, haemathrin wild-type proteins; haemathrin 2S, haemathrin 2 sulfation proteins.

Supplementary figure S2. The effect of sulfated haemathrin on the invasion of SKOV3 and MDA-MB-231 cells using a Transwell cell invasion assay. The statistical graph indicates the density of invaded cells per field, 24 h after seeding. Results are presented as mean +/- standard deviation of three independent experiments performed in triplicate (*$p < 0.05$). SF, serum free; Haemathrin WT, haemathrin wild-type proteins; haemathrin 2S, haemathrin 2 sulfation proteins.
