# Supplementary figures and images for: Inhibition of Cancer Cell Migration and Invasion In Vitro by Recombinant Tyrosine-Sulfated Haemathrin, A Thrombin Inhibitor
Source: Int J Mol Sci. 2024 Nov 4;25(21):11822. doi: 10.3390/ijms252111822 (PMC11546549; doi:10.3390/ijms252111822)

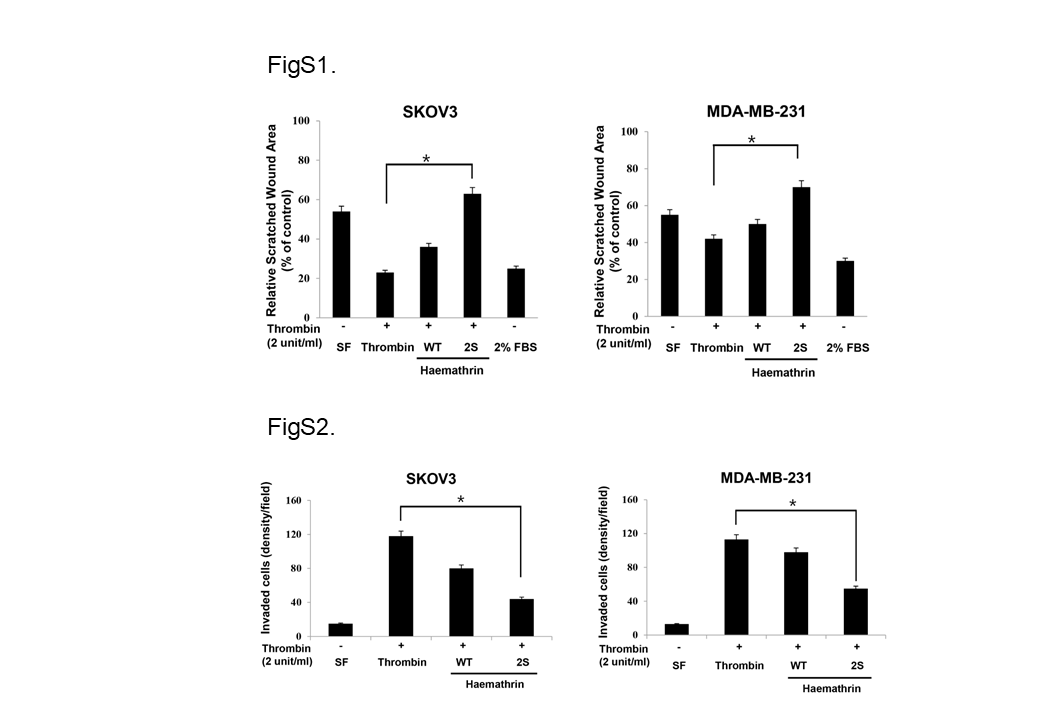

Supplement: Supplementary file 1 [file ijms-25-11822-s001.zip › FigS1 and FigS2.tif]
